# Supplementary material for: Larger language models better align with neural representations of natural language
Source: eLife. 2026 Sep 16;13:RP101204. doi: 10.7554/eLife.101204 (PMC13581289; doi:10.7554/eLife.101204)
Supplement: Peer review history

# Editor report: eLife Assessment

**DOI:** 10.7554/eLife.101204.3.sa0

**Authors:** Nai Ding

**Affiliations:** Zhejiang University China

**Keywords:** Important, Solid

This **important** study investigates how the size of an LLM may influence its ability to model the human neural response to language recorded by ECoG. Overall, **solid** evidence is provided that larger language models can better predict the human ECoG response. This study will be of interest to both neuroscientists and psychologists who work on language comprehension and computer scientists working on LLMs.

# Referee report: Reviewer #1 (Public review)

**DOI:** 10.7554/eLife.101204.3.sa1

Summary:

The authors perform an analysis of the relationship between the size of an LMM and the predictive performance of an ECoG encoding model made using the representations from that LMM. They find a logarithmic relationship between model size and prediction performance, consistent with previous findings in fMRI. They additionally observe that as the model size increases, the location of the "peak" encoding performance typically moves further back into the model in terms of percent layer depth, an interesting result worthy of further analysis into these representations.

Strengths:

The evidence is quite convincing, consistent across model families and complementary to other work in this field. This sort of analysis for ECoG is needed and supports the decade-long enduring trend of the "virtuous cycle" between neuroscience and AI research, where more powerful AI models have consistently yielded more effective predictions of responses in the brain. The lag analysis showing that optimal lags do not change with model size is a nice result using the higher temporal resolution of ECoG compared to other methods like fMRI.

Comments on revised version.

After the latest revision, I am pleased to remove my previous remarks about weaknesses of the paper, as I believe the additional data scaling analysis, discussion of layerwise trends, and other additional commentary makes the paper a compelling addition to the literature.

# Referee report: Reviewer #2 (Public review)

**DOI:** 10.7554/eLife.101204.3.sa2

Summary:

This paper investigates whether large language models (LLMs) of increasing size more accurately align with brain activity during naturalistic language comprehension. The authors extracted word embeddings from LLMs for each word in a 30-minute story and regressed them against electrocorticography (ECoG) activity time-locked to each word as participants listened to the story. The findings reveal that larger LLMs more effectively predict ECoG activity, reflecting the scaling laws observed in other natural language processing tasks.

Strengths:

(1) The study compared model activity with ECoG recordings, which offer much better temporal resolution than other neuroimaging methods, allowing for the examination of model encoding performance across various lags relative to word onset.

(2) The range of LLMs tested is comprehensive, spanning from 82 million to 70 billion parameters. This serves as a valuable reference for researchers selecting LLMs for brain encoding and decoding studies.

(3) The regression methods used are well-established in prior research, and the results demonstrate a convincing scaling law for the brain encoding ability of LLMs. The consistency of these results after PCA dimensionality reduction further supports the claim.

Comments on revised version.

I thank the authors very much for their efforts in addressing my comments. One remaining concern is the extent of the paper's conceptual advance. Several recent studies have made broadly similar claims regarding the increasing alignment between large language models and human language processing, although using fMRI data (Antonello et al., 2023; Gao et al., 2025). I would therefore encourage the authors to more clearly articulate what additional insights are gained from using ECoG. Clarifying this point would help better establish the novelty and contribution of the present study.

Antonello, R. J., Vaidya, A. R., & Huth, A. G. (2023). Scaling laws for language encoding models in fMRI. Advances in Neural Information Processing Systems, 36, 21895-21907.

Gao, C., Ma, Z., Chen, J., Li, P., Huang, S., & Li, J. (2025). Increasing alignment of large language models with language processing in the human brain. Nature Computational Science, 5(11), 1080-1090.

# Referee report: Reviewer #3 (Public review)

**DOI:** 10.7554/eLife.101204.3.sa3

This manuscript studies the connection between neural activity collected through electrocorticography and hidden vector representations from autoregressive language models, with the specific aim of studying the influence of language model size on this connection. Neural activity was measured from subjects that listened to a segment from a podcast, and the representations from language models were calculated using the written transcription as the input text. The ability of vector representations to predict neural activity was evaluated using 10-fold cross-validation with ridge regression models.

The main results are that (as well summarized in section headings):

(1) Larger models predict neural activity better.

(2) The ability of language model representations to predict neural activity differs across electrodes and brain regions.

(3) The layer that best predicts neural activity differs according to model size, with the "SMALL" model showing a correspondence between layer number and the language processing hierarchy.

(4) There seems to be a similar relationship between the time lag and the ability of language model representations to predict neural activity across models.

Strengths:

(1) The experimental and modeling protocols generally seem solid, which yielded results that answer the authors' primary research question.

(2) Electrocorticography data is especially hard to collect, so these results make a nice addition to recent functional magnetic resonance imaging studies.

Weaknesses:

(1) The interpretation of some results seems unjustified, although this may just be a presentational issue.

a) Figure 2B: The authors interpret the results as "a plateau in the maximal encoding performance," when some readers might interpret this rather as a decline after 13 billion parameters. Can this be further supported by a significance test like that shown in Figure 4B?

b) Figure S1A: It looks like the drop in PCA max correlation is larger for larger models, which may suggest to some readers that the same trend observed for ridge max correlation may not hold, contra the authors' claim that all results replicate. Why not include a similar figure as Figure 2B as part of Figure S1?

(2) Discussion of what might be driving the main result about the influence of model size appears to be missing (cf. the authors aim to provide an explanation of what seems to drive the influence of the layer location in Paragraph 3 of the Discussion section). What explanations have been proposed in the previous functional magnetic resonance imaging studies? Do those explanations also hold in the context of this study?

(3) The GloVe-based selection of language-sensitive electrodes (at least to me) isn't explained/motivated clearly enough (I think a more detailed explanation should be included in the Materials and Methods section). If the electrodes are selected based on GloVe embeddings, then isn't the main experiment just showing that representations from larger language models track more closely with GloVe embeddings? What justifies this methodology?

(4) (Minor weakness) The main experiments are largely replications of previous functional magnetic resonance imaging studies, with the exception of the one lag-based analysis. Is there anything else that the electrocorticography data can reveal that functional magnetic resonance imaging data can't?

Comments on revised version.

I reread the manuscript, my previous review, and the authors' response to it. I thank the authors for clarifying any misunderstanding from my end (e.g. the different LLM tokenizers) and feel that the authors addressed my concerns very carefully.

# Author comment: Author response

**DOI:** 10.7554/eLife.101204.3.sa4

**Authors:** Zhuoqiao Hong, Haocheng Wang, Zaid Zada, Harshvardhan Gazula, David Turner, Bobbi Aubrey, Leonard Niekerken, Werner Doyle, Sasha Devore, Patricia Dugan, Daniel Friedman, Orrin Devinsky, Adeen Flinker, Uri Hasson, Samuel Nastase, Ariel Y Goldstein

**Affiliations:** Princeton University Princeton United States; Princeton University Princeton United States; Princeton University Princeton United States; Massachusetts General Hospital Boston United States; Princeton University Princeton United States; Princeton University Princeton United States; Princeton University Princeton United States; New York University School of Medicine New York United States; New York University School of Medicine New York United States; New York University School of Medicine New York United States; New York University School of Medicine New York United States; NYU Langone Health New York City United States; New York University New York United States; Princeton University Princeton United States; Princeton University Princeton United States; Hebrew University of Jerusalem Jerusalem Israel

The following is the authors’ response to the original reviews

**Public Reviews:**

**Reviewer #1 (Public review):**

Summary:

The authors perform an analysis of the relationship between the size of an LMM and the predictive performance of an ECoG encoding model made using the representations from that LMM. They find a logarithmic relationship between model size and prediction performance, consistent with previous findings in fMRI. They additionally observe that as the model size increases, the location of the "peak" encoding performance typically moves further back into the model in terms of percent layer depth, an interesting result worthy of further analysis into these representations.

Strengths:

The evidence is quite convincing, consistent across model families, and complementary to other work in this field. This sort of analysis for ECoG is needed and supports the decade-long enduring trend of the "virtuous cycle" between neuroscience and AI research, where more powerful AI models have consistently yielded more effective predictions of responses in the brain. The lag analysis showing that optimal lags do not change with model size is a nice result using the higher temporal resolution of ECoG compared to other methods like fMRI.

We thank the reviewer for their thoughtful assessment! We agree that the “virtuous cycle” between neuroscience and AI research has been, and will continue to be, a driving force in advancing our understanding of brain function through more powerful predictive models. We are especially pleased that the reviewer appreciated the lag analysis, as we view this as a valuable complement to the existing fMRI work.

Weaknesses:

I would have liked to have seen the data scaling trends explored a bit too, as this is somewhat analogous to the main scaling results. While better performance with more data might be unsurprising, showing good data scaling would be a strong and useful justification for additional data collection in the field, especially given the extremely limited amount of existing language ECoG data. I realize that the data here is somewhat limited (only 30 minutes per subject), but authors could still in principle train models on subsets of this data.

We thank the reviewer for their valuable suggestion. For the revised manuscript, we performed a new analysis where we trained encoding models using subsets of the data (randomly sampling contiguous chunks of 50%, 25%, and 10% of all words in each of the training folds) and tested these models on all words in the test fold. As expected, we found that encoding performance increases as the training dataset size increases, suggesting that model performance scales with data quantity even within the constraints of our relatively small dataset. This result reinforces the importance of collecting dense ECoG data. We have added the following text to our Results section: “We also built encoding models using subsets of the data and found that encoding performance increases as the volume of training data increases (Fig. S6)” and included the results as a supplementary figure 6 in the revised manuscript.

Separately, it would be nice to have better justification of some of these trends, in particular the peak layerwise encoding performance trend and the overall upside-down U-trend of encoding performance across layers more generally. There is clearly something very fundamental going on here, about the nature of abstraction patterns in LLMs and in the brain, and this result points to that. I don't see the lack of justification here as a critical issue, but the paper would certainly be better with some theoretical explanation for why this might be the case.

We thank the reviewer for this insightful comment. The general inverted U-shaped trend of encoding performance across layers has been a frequently observed phenomenon in studies comparing LLM representations to brain activity (Goldstein, Ham, et al., 2025; Schrimpf et al., 2021). A potential explanation is the existence of a “two-phase abstraction process” within LLMs (Cheng & Antonello, 2024; Csordás et al., 2025). In the initial layers, models begin by processing relatively low-level input features. As layers get deeper, representations become increasingly abstract and richly contextualized in semantic features relevant for understanding language. These intermediate layers often show the highest correlation with brain activity in language areas, presumably because they capture complex semantic and contextual information in a way that generalizes well across a variety of tasks (including prediction of human neural activity) (Antonello & Huth, 2024). Subsequently, a prediction phase happens in the later layers, where the representations become more specialized for the LLM's specific training objective (e.g., next-word prediction). This specialization can effectively constrict the more generalized feature representations, making these layers less optimal for predicting brain activity. These observations suggest that it is primarily the abstractive, contextual features developed in the intermediate layers of LLMs that drive their alignment with brain activity. As models become more potent at prediction, their most predictive layers (for the LLM’s natural language task) and their most generalizable layers (for brain activity) can diverge.

A key finding in our study is that the initial processing phase does not scale and take up more layers as models scale up in size and layers. Larger models develop the necessary rich, abstract representations in the same number of layers as smaller models. Consequently, the prediction phase may begin relatively earlier in these larger models, and the later layers could develop highly specialized representations that are increasingly divergent from the more general linguistic processing captured in brain activity. For example, these layers may specialize in capturing very specific patterns of language (thus lowering their perplexity) that do not actually occur often or at all in our naturalistic dataset. It is also possible that the later layers of larger models are overall underutilized and do not contribute as much to linguistic processing and next-word prediction (Csordás et al., 2025).

We have added the following text to our Discussion section:

“The inverted U-shaped trend of encoding performance commonly found in previous research is likely due to a "two-phase abstraction process" within LLMs (Cheng & Antonello, 2024; Csordás et al., 2025). In the early and intermediate layers of the model, a composition phase occurs, where low-level input features become increasingly abstract and contextualized. The intermediate layers of the model show the highest correlation with brain activity, presumably because they capture complex semantic and contextual information in a way that generalizes well across a variety of tasks (including prediction of human neural activity) (Antonello & Huth, 2024). Subsequently, a prediction phase happens in the later layers of the model, where the representations become more specialized for the LLM's specific training objective (e.g., next-word prediction). This specialization can effectively constrict the more generalized feature representations, making these layers less optimal for predicting brain activity. Our results indicate that the initial composition phase does not take up more layers as models scale up in size. Larger models develop the necessary rich, abstract representations in the same number of layers as smaller models. Thus, as LLMs increase in size, the later layers of the model may contain representations that are increasingly divergent from the more general linguistic processing captured in brain activity. It is also possible that the later layers of larger models are overall underutilized and may not significantly contribute to benchmark performances during inference (Csordás et al., 2025; Fan et al., 2024; Gromov et al., 2024).”

Lastly, I would have wanted to see a similar analysis here done for audio encoding models using Whisper or WavLM as this is the modality where you might see real differences between ECoG and other slower scanning approaches. Again, I do not see this omission as a fundamental issue, but it does seem like the sort of analysis for which the higher temporal resolution of ECoG might grant some deeper insight.

We appreciate this suggestion. In a separate project, we focused on multimodal audio-to-speech-to-language large language models (LLMs), building encoding models using Whisper embeddings (from both the encoder and decoder stacks) to predict electrocorticographic (ECoG) signals during naturalistic conversations (Goldstein, Wang, et al., 2025). The higher temporal resolution of ECoG enables us to trace the temporal flow of information from the superior temporal gyrus (STG) and somatomotor areas (SM) to the inferior frontal gyrus (IFG) during speech comprehension. Conversely, during speech production, encoding in IFG peaked significantly earlier than in the STG and SM. We agree that scaling encoding models using multimodal approaches and our ECoG conversation datasets could yield valuable insights, and we look forward to exploring this in future work. However, we feel that the added complexity of multimodal encoding models falls beyond the scope of this paper.

We have modified the following text to our Discussion section:

“Since we exclusively employ textual LLMs, which lack inherent temporal information due to their discrete token-based nature, future studies utilizing multimodal LLMs integrating continuous audio or video streams, like Whisper or WavLM may better unravel the relationship between model size and temporal dynamic representations in LLMs (Goldstein, Wang, et al., 2025; Millet et al., 2023; Vaidya et al., 2022).”

**Reviewer #2 (Public review):**

Summary:

This paper investigates whether large language models (LLMs) of increasing size more accurately align with brain activity during naturalistic language comprehension. The authors extracted word embeddings from LLMs for each word in a 30-minute story and regressed them against electrocorticography (ECoG) activity time-locked to each word as participants listened to the story. The findings reveal that larger LLMs more effectively predict ECoG activity, reflecting the scaling laws observed in other natural language processing tasks.

Strengths:

(1) The study compared model activity with ECoG recordings, which offer much better temporal resolution than other neuroimaging methods, allowing for the examination of model encoding performance across various lags relative to word onset.

(2) The range of LLMs tested is comprehensive, spanning from 82 million to 70 billion parameters. This serves as a valuable reference for researchers selecting LLMs for brain encoding and decoding studies.

(3) The regression methods used are well-established in prior research, and the results demonstrate a convincing scaling law for the brain encoding ability of LLMs. The consistency of these results after PCA dimensionality reduction further supports the claim.

We thank the reviewer for their thoughtful and positive feedback.

Weaknesses:

(1) Some claims of the paper are less convincing. The authors suggested that "scaling could be a property that the human brain, similar to LLMs, can utilize to enhance performance", however, many other animals have brains with more neurons than the human brain, making it unlikely that simple scaling alone leads to better language performance.

We thank the reviewer for this insightful comment. We agree that simply having more neurons does not automatically confer more complex or human-like cognitive or linguistic capabilities. This suggestion deserves a more nuanced treatment than we had included in the original manuscript.

Research in comparative neuroscience has argued that human cognitive abilities emerge from scaling up the primate brain (Herculano-Houzel, 2012). However, the critical aspect is not merely the number of neurons, but how these neurons contribute to computational power within a specific evolutionary and cultural context. The uniqueness of human cognition has been argued to result from a global adaptation for increased information processing capacity (Cantlon & Piantadosi, 2024). Moreover, the language network in humans is likely grounded in the evolution of particular structural networks in the primate brain (Friederici & Becker, 2025). This suggests that the way brain regions are connected and the expansion of certain pathways are critical, not just the overall scale. Furthermore, the specialized structure must be tuned by its learning environment and training data. For example, both humans and LLMs learn from language data generated by other humans, which reflects world knowledge that has accumulated over many generations.

We have modified the following text in the Introduction:

“Research in comparative neuroscience has suggested that uniquely human cognitive abilities emerge from scaling up the primate brain (Herculano-Houzel, 2012).”

We also added a caveat to the Discussion on this point:

“As in the human brain, while scaling alone may yield emergent cognitive abilities (Cantlon & Piantadosi, 2024; Herculano-Houzel, 2012), specialized architectural features likely also play a critical role (Friederici & Becker, 2025).”

Additionally, the authors claim that their results show 'larger models better predict the structure of natural language.' However, it remains unclear to what extent the embeddings of LLMs capture the "structure" of language better than the lexical semantics of language.

We appreciate the reviewer's point about how well LLM embeddings capture the "structure" of language versus just lexical semantics. It's true that distinguishing these aspects is complex. From our perspective, a model's ability to predict/produce natural language entails that the model captures various levels of linguistic structure, including morphology, syntax, semantics, and contextual dependencies. We use "structure" inclusively in this sense. A model cannot achieve high predictive accuracy without representing, to some extent, all of these structural elements (Linzen & Baroni, 2021; Manning et al., 2020; Pavlick, 2022). There is a very active field of research into understanding exactly *how* these models represent these different structures of language (Ameisen et al., 2025; Chemla et al., 2024; Elhage et al., 2021, 2022; Hewitt & Manning, 2019). Our results confirm the core trend that larger models tend to better reproduce the various structures of language (i.e., yield lower perplexity; Fig. 2A).

In previous work, we have shown that LLM embeddings better predict neural activity during natural language processing than lexical embeddings (e.g., GloVe) that do not contain other elements of linguistic structure (Goldstein et al., 2022; Kumar et al., 2024; Zada et al., 2024). In response to the following comment, we also compare LLMs to simpler models capturing specific speech and language features (see next comment). To clarify our intended use of the word “structure”, we’ve added a brief explanation in the Methods section:

“In this study, we use the term “structure” to refer to a variety of linguistic patterns (e.g., morphology, syntax, semantics, context) that LLMs encode in order to better predict natural language.”

(2) The study lacks control LLMs with randomly initialized weights and control regressors, such as word frequency and phonetic features of speech, making it unclear what the baseline is for the model-brain correlation.

We’ve added several supplementary analyses to the revised manuscript to address these concerns. To establish a baseline, we extracted embeddings from each layer of the SMALL model with randomly initialized weights and constructed encoding models. The encoding performance is significantly higher for pretrained SMALL than for untrained SMALL for every layer (Fig. S4). For the untrained model, the performance is the highest for the 0th layer and decreases in subsequent layers. This is because at the 0th layer, every instance of the same word receives an identical, albeit random, embedding (See Supplementary Figure 4).

We also compared the encoding performance of LLMs with more classical speech/language features and static GloVe embeddings (Goldstein, Wang, et al., 2025; Kumar et al., 2024). First, we extracted features capturing lower-level speech features. Using the stimulus transcript as input, we created one-hot vectors for phonetic and articulatory features. Phoneme classes (39 total classes) were obtained from the Carnegie Mellon Pronouncing Dictionary (The CMU Pronouncing Dictionary, n.d.). We further classified the phonemes based on their place of articulation (9 classes), manner of articulation (9 classes), and voiced or voiceless status (3 classes), according to the general American English consonants of the International Phonetic Alphabet. Given that each word consists of multiple phonemes, we averaged the one-hot vectors for all phonetic and articulatory features for each word.

Second, we extracted linguistic features using spaCy (Honnibal et al., 2020), including part of speech (17 classes), tag (50 classes), function or content word (3 classes), dependency (45 classes), whether the word is an alpha character (binary), and whether the word is a stop word (binary). We also extracted prefix (30 classes) and suffix (44 classes) information using the Cambridge Dictionary. We constructed one-hot vectors for each multi-class feature and one-dimensional vectors for each binary feature.

Third, for each word, we obtained word frequency from the Google Web Trillion Word Corpus (Brants & Franz, 2006) and from our own dataset.

Fourth, we generated static word embeddings of dimension 50 using GloVe (Pennington et al., 2014).

We then built encoding models in the same way as the contextual embeddings for each of the three categories of speech features, all speech features concatenated, and the GloVe embeddings. To control for the different dimensions of the embeddings, we also standardized all embeddings to the same size (50 dimensions) using principal component analysis (PCA) and trained linear encoding models using ordinary least-squares (OLS) regression. For both ridge and OLS encoding, our contextual embeddings from LLMs showed significantly better performance than the classic speech features and GloVe embeddings.

We have added the following text to our manuscript and updated our Figures S4, S5, Table S1, and the methods section:

“To establish a general baseline for encoding performance, we built encoding models using embeddings from the SMALL model with randomly initialized weights. The trained SMALL model exhibits significantly higher encoding performance across all layers compared to the untrained SMALL model (Fig. S4). We also assessed the encoding performance of contextual embeddings from LLMs against classic speech features and static GloVe embeddings (Table S1). The SMALL and XL embeddings achieved markedly higher encoding correlations than the speech features and GloVe embeddings (Fig. S5).”

(3) The finding that peak encoding performance tends to occur in relatively earlier layers in larger models is somewhat surprising and requires further explanation. Since more layers mean more parameters, if the later layers diverge from language processing in the brain, it raises the question of what aspects of the larger models make them more brain-like.

We thank the reviewer for this insightful comment; this point was also highlighted by Reviewer 1. We agree that this result is somewhat surprising, and we aim to provide a more detailed explanation in the revised manuscript. The general inverted U-shaped trend of encoding performance across layers has been a frequently observed phenomenon in studies comparing LLM representations to brain activity (Goldstein, Ham, et al., 2025; Schrimpf et al., 2021). A potential explanation is the existence of a “two-phase abstraction process” within LLMs (Cheng & Antonello, 2024; Csordás et al., 2025). In the initial layers, models begin by processing relatively low-level input features. As layers get deeper, representations become increasingly abstract and richly contextualized in semantic features relevant for understanding language. These intermediate layers often show the highest correlation with brain activity in language areas, presumably because they capture complex semantic and contextual information in a way that generalizes well across a variety of tasks (including prediction of human neural activity) (Antonello & Huth, 2024). Subsequently, a prediction phase happens in the later layers, where the representations become more specialized for the LLM's specific training objective (e.g., next-word prediction). This specialization can effectively constrict the more generalized feature representations, making these layers less optimal for predicting brain activity. These observations suggest that it is primarily the abstractive, contextual features developed in the intermediate layers of LLMs that drive their alignment with brain activity. As models become more potent at prediction, their most predictive layers (for the LLM’s natural language task) and their most generalizable layers (for brain activity) can diverge.

A key finding in our study is that the initial processing phase does not scale and take up more layers as models scale up in size and layers. Larger models develop the necessary rich, abstract representations in the same number of layers as smaller models.

Consequently, the prediction phase may begin relatively earlier in these larger models, and the later layers could develop highly specialized representations that are increasingly divergent from the more general linguistic processing captured in brain activity. For example, these layers may specialize in capturing very specific patterns of language (thus lowering their perplexity) that do not actually occur often or at all in our naturalistic dataset. It is also possible that the later layers of larger models are overall underutilized and do not contribute as much to linguistic processing and next-word prediction (Csordás et al., 2025).

We have added the following text to our Discussion section:

“The inverted U-shaped trend of encoding performance commonly found in previous research is likely due to a "two-phase abstraction process" within LLMs (Cheng & Antonello, 2024; Csordás et al., 2025). In the early and intermediate layers of the model, a composition phase occurs, where low-level input features become increasingly abstract and contextualized. The intermediate layers of the model show the highest correlation with brain activity, presumably because they capture complex semantic and contextual information in a way that generalizes well across a variety of tasks (including prediction of human neural activity) (Antonello & Huth, 2024). Subsequently, a prediction phase happens in the later layers of the model, where the representations become more specialized for the LLM's specific training objective (e.g., next-word prediction). This specialization can effectively constrict the more generalized feature representations, making these layers less optimal for predicting brain activity. Our results indicate that the initial composition phase does not take up more layers as models scale up in size. Larger models develop the necessary rich, abstract representations in the same number of layers as smaller models. Thus, as LLMs increase in size, the later layers of the model may contain representations that are increasingly divergent from the more general linguistic processing captured in brain activity. It is also possible that the later layers of larger models are overall underutilized and may not significantly contribute to benchmark performances during inference (Csordás et al., 2025; Fan et al., 2024; Gromov et al., 2024).”

**Reviewer #3 (Public review):**

This manuscript studies the connection between neural activity collected through electrocorticography and hidden vector representations from autoregressive language models, with the specific aim of studying the influence of language model size on this connection. Neural activity was measured from subjects who listened to a segment from a podcast, and the representations from language models were calculated using the written transcription as the input text. The ability of vector representations to predict neural activity was evaluated using 10-fold cross-validation with ridge regression models.

The main results are that (as well summarized in section headings):

(1) Larger models predict neural activity better.

(2) The ability of language model representations to predict neural activity differs across electrodes and brain regions.

(3) The layer that best predicts neural activity differs according to model size, with the "SMALL" model showing a correspondence between layer number and the language processing hierarchy.

(4) There seems to be a similar relationship between the time lag and the ability of language model representations to predict neural activity across models.

Strengths:

(1) The experimental and modeling protocols generally seem solid, which yielded results that answer the authors' primary research question.

(2) Electrocorticography data is especially hard to collect, so these results make a nice addition to recent functional magnetic resonance imaging studies.

We thank the reviewer for their thoughtful and positive feedback.

Weaknesses:

(1) The interpretation of some results seems unjustified, although this may just be a presentational issue.

(a) Figure 2B: The authors interpret the results as "a plateau in the maximal encoding performance," when some readers might interpret this rather as a decline after 13 billion parameters. Can this be further supported by a significance test like that shown in Figure 4B?

We agree that this could be a subjective interpretation, so we conducted an additional analysis. We performed paired two-sided *t*-tests between best layer encoding performances averaged across electrodes (df = 159 electrodes), comparing all models with larger models. We found that after 13 billion parameters, only the encoding performance for OPT-66B, the largest model in the OPT family, is significantly worse than the encoding performance of some other smaller models, supporting the claim that the maximal encoding performance declines after 13 billion parameters. However, we did not find conclusive statistical evidence of a decline in encoding performance for other model families.

We have added the statistical results as Supplementary Figure 1.

We have also modified the following text in the manuscript:

“We also observed a plateau in the maximal encoding performance, occurring around 7 billion parameters (Fig. 2B), with a decline in performance for the OPT-66B model (Fig. S1).”

(b) Figure S1A: It looks like the drop in PCA max correlation is larger for larger models, which may suggest to some readers that the same trend observed for ridge max correlation may not hold, contra the authors' claim that all results replicate. Why not include a similar figure as Figure 2B as part of Figure S1?

PCA is an unsupervised dimensionality reduction technique and may discard model features with small eigenvalues that nonetheless contribute to encoding performance. Ridge regression, a supervised method, can capitalize on these features. We suspect that this is why there appears to be a larger drop in model performance for larger models with PCA than with ridge regression. We replicated the logarithmic relationship between model size and encoding performance using PCA and ordinary least-squares (OLS) regression encoding models. We have updated Supplementary Figure 2.

(2) Discussion of what might be driving the main result about the influence of model size appears to be missing (cf. the authors aim to provide an explanation of what seems to drive the influence of the layer location in Paragraph 3 of the Discussion section). What explanations have been proposed in the previous functional magnetic resonance imaging studies? Do those explanations also hold in the context of this study?

We suspect that the increased expressivity of larger models - that is, their improved sensitivity to nuanced structure in natural language - yields improved alignment to brain activity (given large enough samples of brain activity) (Antonello et al., 2023). This effect persists even when dimensionality is tightly controlled in our PCA-based analysis, indicating that the improved alignment with the brain is not a modeling artifact of dimensionality alone, but results from the structural representations learned by these larger models.

We have added the following text to our Discussion section:

“We suspect that the improved alignment with brain activity in larger models is driven by their increased expressivity and sensitivity to nuanced linguistic structure present in large-scale naturalistic datasets (Antonello et al., 2023).”

(3) The GloVe-based selection of language-sensitive electrodes (at least to me) isn't explained/motivated clearly enough (I think a more detailed explanation should be included in the Materials and Methods section). If the electrodes are selected based on GloVe embeddings, then isn't the main experiment just showing that representations from larger language models track more closely with GloVe embeddings? What justifies this methodology?

We selected electrodes based on previously established methods (Goldstein et al., 2022). Our use of GloVe embeddings for electrode selection does not imply that larger language model representations are simply more closely aligned with GloVe embeddings. On the contrary, contextual embeddings from LLMs, which incorporate the word’s previous context, consistently outperform static embeddings like GloVe or word2vec (Fig. S3). Selecting electrodes using LLM embeddings would likely result in a slightly different, potentially larger set of electrodes (Goldstein et al., 2022), but would be more circular (Kriegeskorte et al., 2009). The GloVe-based electrode selection represents a more conservative approach by identifying words encoding linguistic content without biasing the selection directly toward any LLMs.

We have added the following text to our Method section:

“We used GloVe embeddings for electrode selection to avoid biasing our main results toward a particular LLM.”

(4) (Minor weakness) The main experiments are largely replications of previous functional magnetic resonance imaging studies, with the exception of the one lag-based analysis. Is there anything else that the electrocorticography data can reveal that functional magnetic resonance imaging data can't?

We thank the reviewer for this thoughtful question. While we agree that a key contribution of our work corroborates previous fMRI findings, we would argue that using ECoG is not merely a replication but a crucial validation and extension of that work. It is important to validate these effects across distinct measurement modalities. In our work, we further observed a novel trend where the peak encoding performance tends to occur in relatively earlier layers for larger models. This is supported by recent studies suggesting that later layers of large LLMs may not significantly contribute to benchmark performance (Csordás et al., 2025). While scaling has been an effective method to improve LLM performance, including in encoding models, future research should explore the potential underutilization of the later layers as models scale.

Furthermore, ECoG data offers temporal resolution on the order of milliseconds, far superior to fMRI’s. Although we did not observe a relationship between model size and temporal lags in this study, future work should investigate the temporal dynamics of encoding that are accessible with ECoG (Goldstein, Ham, et al., 2025; Goldstein, Wang, et al., 2025).

**Recommendations for the authors:**

**Reviewer #1 (Recommendations for the authors):**

Thank you to the authors for the fun and personally useful read.

I see in Supplementary Figure 1 the authors show a comparison of the performance between OLS vs. Ridge regression. Is the OLS model the only one that is working over PC features, or are both models using PC features? The current text is a bit unclear. My current understanding is that the comparison is between (OLS + PCA) and (Ridge with no PCA), but I am not sure.

The OLS model is the only one that works over PC features, following previous methods (Goldstein et al., 2022).

We have added the following text to our Results and Methods section for clarity:

“To control for the different embedding dimensionality across models, we standardized all embeddings to the same size using principal component analysis (PCA) and trained linear encoding models using ordinary least-squares (OLS) regression, replicating the logarithmic relationship but with significantly lower encoding performance overall (Fig. S2). The PC features are used by the OLS models only.”

Clarification in the text would be appropriate. If this is the correct understanding, the authors should note in the main text that the ridge approach is more effective than the PCA approach, which is still the dominant approach to building linear encoding models in the field for some unjustifiable reason.

We thank the reviewer for pointing out the confusion. We have updated Supplementary Figure 2.

How were the alpha values for ridge regression determined? Do you use the same ridge parameter for all electrodes or fit a different parameter for each electrode? This is not mentioned anywhere.

The alpha values are determined by cross-validation using the “RidgeCV” method from the “himalaya” package (Dupré la Tour et al., 2022). Specifically, we perform a grid search over cross-validation folds in the training data to find the best-performing alpha. The alpha parameter is specific to each ridge regression model, meaning each fold, lag, and electrode has a different alpha parameter.

We have added the following text to our manuscript:

“For each ridge regression model (for each fold, lag, and electrode), the alpha parameter is determined by cross-validation using the “RidgeCV” method from the “himalaya” package (Dupré la Tour et al., 2022).”

It's not entirely clear to me how the authors handle tokens that do not terminate in words (such as the "there" + "'s" example in the text). My current reading of the text is that authors essentially ignore these half-word embeddings, doing one forward pass per word, rather than per token, but the current text is somewhat ambiguous.

If a word is tokenized into several tokens, like “there” and “‘s”, we average the token embeddings to get a word embedding.

We have added the following text to our Method section:

“To facilitate a fair comparison of the encoding effect across different models, we aligned all tokens in the story across all models. We averaged the token embeddings if a word is split into multiple tokens, resulting in one embedding per word for each model.”

The authors describe the scaling relationship they find as a "log-linear" relationship. I believe this is a misnomer derived from the original paper describing this relationship in fMRI as log-linear (Antonello et al.) The correct term is simply "logarithmic", and for what it's worth, the authors of the original fMRI work have made this correction as well.

Thank you! We have made this correction.

Is the data publicly available? If not, there should be some basic justification as to why (consent reasons, etc.).

We have recently made the data publicly available (Zada et al., 2025). We have also provided tutorials for preprocessing the data and training encoding models: here. For this specific project, the analysis code is available at here and here.

The authors assert that ECoG has "superior spatiotemporal resolution". While this is unquestionably true for temporal resolution, the story is a bit more complicated for spatial resolution, where ECoG has far less cortical coverage than fMRI. Perhaps this sentence should be revised.

Thank you for pointing out the typo! We have changed it to “superior temporal resolution”.

Minor Points:

The bolded title of Figure 3 probably shouldn't be bolded, as this is just actually the title of Figure 3A.

Fixed.

Figure 4d is has a typo: "Best Encoidng Layer".

Fixed.

**Reviewer #2 (Recommendations for the authors):**

The authors could consider adding control regressors such as word rate, word frequency, phonetic features, and syntactic features like node counts, as well as control LLMs of comparable size to serve as baselines. The authors could also include correlation analyses of the embeddings from different layers of the same LLM to further illustrate how distinct the layers are within the models.

We have added untrained LLM embeddings as a baseline and included a comparison of encoding models between LLM contextual embeddings and classical speech features. We have also performed some preliminary correlation analyses of embeddings. In some models, we found evidence of the “two-phase abstraction process” (Cheng & Antonello, 2024). However, the result is inconclusive across different LLM families. Since each LLM layer accesses and modifies the residual stream (Elhage et al., 2021), the embeddings across layers are inherently correlated. Future work could instead explore the isolated transformations within each layer to illustrate the distinct information across layers (Kumar et al., 2024).

The analysis codes and data should be made available.

We have recently made the data publicly available (Zada et al., 2025). We have also provided tutorials for preprocessing the data and training encoding models: here. For this specific project, the analysis code is available at here and here.

**Reviewer #3 (Recommendations for the authors):**

Most of my concrete recommendations are in the public review. Below are some additional minor ones:

(1) Introduction: "Remarkably, these models learn from much the same shared space as humans: from real-world language generated by humans."

I think this is an extremely strong claim due to e.g. the different nature of child-directed speech vs. written text corpora, the lack of multimodality and grounding in language models, etc. I might suggest re-wording this sentence or removing it entirely.

We thank the reviewer for their suggestion! We have removed the sentence from the manuscript.

(2) Introduction: "EleutherAI, n.d." reference for GPT-Neo

GPT-NeoX-20B has an associated paper, which the authors might cite instead: https://aclanthology.org/2022.bigscience-1.9

Thank you! We have added the reference for GPT-NeoX-20B (Black et al., 2022).

(3) Figure 4D: Encoidng -> Encoding

Fixed.

(4) Materials and Methods, Contextual embeddings: "except for GPT-Neox-20b, which assigns additional tokens to whitespace characters."

What do the authors mean by "additional tokens to whitespace characters?" The tokenizer for GPT-NeoX-20B works in much the same way as that of GPT-Neo, just with a different vocabulary set.

>>> t1 = AutoTokenizer.from_pretrained("EleutherAI/gpt-neo-125M")

>>> t2 = AutoTokenizer.from_pretrained("EleutherAI/gpt-neox-20b")

>>> t1.convert_ids_to_tokens(t1("The quick brown fox jumps over the lazy dog.").input_ids) ['The', 'Ġquick', 'Ġbrown', 'Ġfox', 'Ġjumps', 'Ġover', 'Ġthe', 'Ġlazy', 'Ġdog', '.']

>>> t2.convert_ids_to_tokens(t2("The quick brown fox jumps over the lazy dog.").input_ids)

['The', 'Ġquick', 'Ġbrown', 'Ġfox', 'Ġjumps', 'Ġover', 'Ġthe', 'Ġlazy', 'Ġdog', '.']

If the authors are referring to Ġ as the "additional token to whitespace characters," then these are in all other tokenizers as well (not only that for GPT-Neo, but also those for GPT-2 and OPT).

We agree that “additional tokens to whitespace characters” is an oversimplification. The GPT-Neo model family, which includes the 125M, 1.3B, and 2.7B models, utilizes the same Byte Pair Encoding (BPE) tokenizer as GPT-2. This common tokenizer has a vocabulary size of 50,257 tokens, providing compatibility and seamless integration across the models.

The GPT-NeoX-20B model introduces a modified tokenizer to address limitations observed in the GPT-2 tokenizer (Black et al., 2022). As detailed in Section 3.2, this new tokenizer incorporates a few key improvements:

(1) New BPE tokenizer: A more general-purpose BPE tokenizer was trained using the Pile dataset.

(2) Space Delimitation: Unlike the GPT-2 tokenizer, which treats tokenization at the start of a string as a non-space-delimited token, the GPT-NeoX-20B tokenizer applies consistent space delimitation regardless. This change resolves inconsistencies related to the presence of prefix spaces in the tokenization input.

(3) Whitespace Handling: The tokenizer includes tokens for repeated space characters (up to 24 consecutive spaces), enhancing efficiency in tokenizing text with substantial whitespace, such as program source code or LaTeX documents.

These modifications result in the GPT-NeoX-20B tokenizer representing the Pile validation set with approximately 10% fewer tokens than the GPT-2 tokenizer. This efficiency gain is particularly beneficial for processing texts with extensive whitespace.

In our analysis, we extracted embeddings by setting `add_prefix_space = True` to all tokenizers, so space delimitation does not result in tokenizer differences. We highlight here examples of the other two tokenizer differences using the Huggingface `AutoTokenizer`:

>>> t1 = AutoTokenizer.from_pretrained("EleutherAI/gpt-neo-125M")

>>> t2 = AutoTokenizer.from_pretrained("EleutherAI/gpt-neox-20b")

>>> t1.convert_ids_to_tokens(t1("The Downing Street.").input_ids) ['The', 'ĠDowning', 'ĠStreet']

>>> t2.convert_ids_to_tokens(t2("The Downing Street.").input_ids)

['The', 'ĠDown', 'ing', 'ĠStreet']

>>> t1.convert_ids_to_tokens(t1("Hello !").input_ids)

['Hello', 'Ġ', 'Ġ', 'Ġ', 'Ġ', 'Ġ', 'Ġ', 'Ġ!']

>>> t2.convert_ids_to_tokens(t2("Hello !").input_ids)

['Hello', ' ', '!']

More examples showing the differences between the GPT-2 tokenizer and the GPT-NeoX-20B tokenizer can be found in Appendix F: Tokenizer Analysis (Black et al., 2022).

We have added the following text to our manuscript for simplicity:

“All models within the same model family adhere to the same tokenizer convention, except for GPT-Neox-20B, which utilizes a different tokenizer (Black et al., 2022).”

References

Ameisen, E., Lindsey, J., Pearce, A., Gurnee, W., Turner, N. L., Chen, B., Citro, C., Abrahams, D., Carter, S., Hosmer, B., Marcus, J., Sklar, M., Templeton, A., Bricken, T., McDougall, C., Cunningham, H., Henighan, T., Jermyn, A., Jones, A., … Batson, J. (2025). Circuit Tracing: Revealing Computational Graphs in Language Models. Transformer Circuits Thread. https://transformer-circuits.pub/2025/attribution-graphs/methods.html

Antonello, R., & Huth, A. (2024). Predictive coding or just feature discovery? An alternative account of why language models fit brain data. Neurobiology of Language (Cambridge, Mass.), 5(1), 64–79.

Antonello, R., Vaidya, A., & Huth, A. G. (2023). Scaling laws for language encoding models in fMRI. NeurIPS 2023. https://doi.org/10.48550/ARXIV.2305.11863

Black, S., Biderman, S., Hallahan, E., Anthony, Q., Gao, L., Golding, L., He, H., Leahy, C., McDonell, K., Phang, J., Pieler, M., Prashanth, U. S., Purohit, S., Reynolds, L., Tow, J., Wang, B., & Weinbach, S. (2022). GPT-NeoX-20B: An Open-Source Autoregressive Language Model. Proceedings of BigScience Episode #5 -- Workshop on Challenges & Perspectives in Creating Large Language Models. Proceedings of BigScience Episode #5 -- Workshop on Challenges & Perspectives in Creating Large Language Models, virtual+Dublin. https://doi.org/10.18653/v1/2022.bigscience-1.9

Brants, T., & Franz, A. (2006). Web 1T 5-gram Version 1 [Dataset]. Linguistic Data Consortium. https://doi.org/10.35111/CQPA-A498

Cantlon, J. F., & Piantadosi, S. T. (2024). Uniquely human intelligence arose from expanded information capacity. Nature Reviews Psychology, 3(4), 275–293.

Chemla, E., D’Ascoli, S., Diego-Simón, P., King, J.-R., & Lakretz, Y. (2024). A Polar coordinate system represents syntax in large language models. Advances in Neural Information Processing Systems 37, 105375–105396.

Cheng, E., & Antonello, R. J. (2024). Evidence from fMRI supports a two-phase abstraction process in language models. In arXiv [cs.CL]. arXiv. http://arxiv.org/abs/2409.05771

Csordás, R., Manning, C. D., & Potts, C. (2025). Do language models use their depth efficiently? In arXiv [cs.LG]. https://doi.org/10.48550/ARXIV.2505.13898

Dupré la Tour, T., Eickenberg, M., Nunez-Elizalde, A. O., & Gallant, J. L. (2022). Feature-space selection with banded ridge regression. In bioRxiv. https://doi.org/10.1101/2022.05.05.490831

Elhage, N., Hume, T., Olsson, C., Schiefer, N., Henighan, T., Kravec, S., Hatfield-Dodds, Z., Lasenby, R., Drain, D., Chen, C., Grosse, R., McCandlish, S., Kaplan, J., Amodei, D., Wattenberg, M., & Olah, C. (2022). Toy Models of Superposition. Transformer Circuits Thread.

Elhage, N., Nanda, N., Olsson, C., Henighan, T., Joseph, N., Mann, B., Askell, A., Bai, Y., Chen, A., Conerly, T., DasSarma, N., Drain, D., Ganguli, D., Hatfield-Dodds, Z., Hernandez, D., Jones, A., Kernion, J., Lovitt, L., Ndousse, K., … Olah, C. (2021). A Mathematical Framework for Transformer Circuits. Transformer Circuits Thread.

Fan, S., Jiang, X., Li, X., Meng, X., Han, P., Shang, S., Sun, A., Wang, Y., & Wang, Z. (2024). Not all Layers of LLMs are Necessary during Inference. In arXiv [cs.CL]. arXiv. http://arxiv.org/abs/2403.02181

Friederici, A. D., & Becker, Y. (2025). The core language network separated from other networks during primate evolution. Nature Reviews. Neuroscience, 26(2), 131–132.

Goldstein, A., Ham, E., Schain, M., Nastase, S. A., Aubrey, B., Zada, Z., Grinstein-Dabush, A., Gazula, H., Feder, A., Doyle, W., Devore, S., Dugan, P., Friedman, D., Brenner, M., Hassidim, A., Matias, Y., Devinsky, O., Siegelman, N., Flinker, A., … Hasson, U. (2025). Temporal structure of natural language processing in the human brain corresponds to layered hierarchy of large language models. Nature Communications, 16(1), 10529.

Goldstein, A., Wang, H., Niekerken, L., Schain, M., Zada, Z., Aubrey, B., Sheffer, T., Nastase, S. A., Gazula, H., Singh, A., Rao, A., Choe, G., Kim, C., Doyle, W., Friedman, D., Devore, S., Dugan, P., Hassidim, A., Brenner, M., … Hasson, U. (2025). A unified acoustic-to-speech-to-language embedding space captures the neural basis of natural language processing in everyday conversations. Nature Human Behaviour. https://doi.org/10.1038/s41562-025-02105-9

Goldstein, A., Zada, Z., Buchnik, E., Schain, M., Price, A., Aubrey, B., Nastase, S. A., Feder, A., Emanuel, D., Cohen, A., Jansen, A., Gazula, H., Choe, G., Rao, A., Kim, C., Casto, C., Fanda, L., Doyle, W., Friedman, D., … Hasson, U. (2022). Shared computational principles for language processing in humans and deep language models. Nature Neuroscience, 25(3), 369–380.

Gromov, A., Tirumala, K., Shapourian, H., Glorioso, P., & Roberts, D. A. (2024). The Unreasonable Ineffectiveness of the Deeper Layers. In arXiv [cs.CL]. arXiv. http://arxiv.org/abs/2403.17887

Herculano-Houzel, S. (2012). The remarkable, yet not extraordinary, human brain as a scaled-up primate brain and its associated cost. Proceedings of the National Academy of Sciences of the United States of America, 109 Suppl 1(supplement_1), 10661–10668.

Hewitt, J., & Manning, C. D. (2019). A Structural Probe for Finding Syntax in Word Representations. In J. Burstein, C. Doran, & T. Solorio (Eds.), Proceedings of the 2019 Conference of the North (pp. 4129–4138). Association for Computational Linguistics.

Honnibal, M., Montani, I., Van Landeghem, S., & Boyd, A. (2020). spaCy: Industrial-strength Natural Language Processing in Python.

Kriegeskorte, N., Simmons, W. K., Bellgowan, P. S. F., & Baker, C. I. (2009). Circular analysis in systems neuroscience: the dangers of double dipping. Nature Neuroscience, 12(5), 535–540.

Kumar, S., Sumers, T. R., Yamakoshi, T., Goldstein, A., Hasson, U., Norman, K. A., Griffiths, T. L., Hawkins, R. D., & Nastase, S. A. (2024). Shared functional specialization in transformer-based language models and the human brain. Nature Communications, 15(1), 5523.

Linzen, T., & Baroni, M. (2021). Syntactic Structure from Deep Learning. Annual Review of Linguistics, 7(1), 195–212.

Manning, C. D., Clark, K., Hewitt, J., Khandelwal, U., & Levy, O. (2020). Emergent linguistic structure in artificial neural networks trained by self-supervision. Proceedings of the National Academy of Sciences of the United States of America, 117(48), 30046–30054.

Millet, J., Caucheteux, C., Orhan, P., Boubenec, Y., Gramfort, A., Dunbar, E., Pallier, C., & King, J.-R. (2023). Toward a realistic model of speech processing in the brain with self-supervised learning. NeurIPS 2022. https://doi.org/10.48550/ARXIV.2206.01685

Pavlick, E. (2022). Semantic structure in deep learning. Annual Review of Linguistics, 8(1), 447–471.

Pennington, J., Socher, R., & Manning, C. (2014). Glove: Global vectors for word representation. Proceedings of the 2014 Conference on Empirical Methods in Natural Language Processing (EMNLP). Proceedings of the 2014 Conference on Empirical Methods in Natural Language Processing (EMNLP), Doha, Qatar. https://doi.org/10.3115/v1/d14-1162

Schrimpf, M., Blank, I. A., Tuckute, G., Kauf, C., Hosseini, E. A., Kanwisher, N., Tenenbaum, J. B., & Fedorenko, E. (2021). The neural architecture of language: Integrative modeling converges on predictive processing. Proceedings of the National Academy of Sciences of the United States of America, 118(45), e2105646118.

The CMU Pronouncing Dictionary. (n.d.). Retrieved May 27, 2025, from http://www.speech.cs.cmu.edu/cgi-bin/cmudict

Vaidya, A. R., Jain, S., & Huth, A. G. (2022). Self-supervised models of audio effectively explain human cortical responses to speech. ICML 2022. https://doi.org/10.48550/ARXIV.2205.14252

Zada, Z., Goldstein, A., Michelmann, S., Simony, E., Price, A., Hasenfratz, L., Barham, E., Zadbood, A., Doyle, W., Friedman, D., Dugan, P., Melloni, L., Devore, S., Flinker, A., Devinsky, O., Nastase, S. A., & Hasson, U. (2024). A shared model-based linguistic space for transmitting our thoughts from brain to brain in natural conversations. Neuron, S0896627324004604. Zada, Z., Nastase, S. A., Aubrey, B., Jalon, I., Michelmann, S., Wang, H., Hasenfratz, L., Doyle, W., Friedman, D., Dugan, P., Melloni, L., Devore, S., Flinker, A., Devinsky, O., Goldstein, A., & Hasson, U. (2025). The “Podcast” ECoG dataset for modeling neural activity during natural language comprehension. Scientific Data, 12(1), 1135.
